# Wafer-scale high-resolution patterning of reduced graphene oxide films for detection of low concentration biomarkers in plasma

**DOI:** 10.1038/srep31276

**Published:** 2016-08-10

**Authors:** Jinsik Kim, Myung-Sic Chae, Sung Min Lee, Dahye Jeong, Byung Chul Lee, Jeong Hoon Lee, YoungSoo Kim, Suk Tai Chang, Kyo Seon Hwang

**Affiliations:** 1 Center for BioMicrosystems, Korea Institute of Science and Technology, Seoul 02792, Korea; 2 School of Chemical Engineering and Materials Science, Chung-Ang University, Seoul 06974, Korea; 3 Department of Electrical Engineering, Kwangwoon University, 447-1 Wolgye, Nowon, Seoul 01897, Korea; 4 Convergence Center for Dementia, Korea Institute of Science and Technology, Seoul 02792, Korea

## Abstract

Given that reduced graphene oxide (rGO)-based biosensors allow disposable and repeatable biomarker detection at the point of care, we developed a wafer-scale rGO patterning method with mass productivity, uniformity, and high resolution by conventional micro-electro-mechanical systems (MEMS) techniques. Various rGO patterns were demonstrated with dimensions ranging from 5 μm up to several hundred μm. Manufacture of these patterns was accomplished through the optimization of dry etching conditions. The axis-homogeneity and uniformity were also measured to verify the uniform patternability in 4-inch wafer with dry etching. Over 66.2% of uniform rGO patterns, which have deviation of resistance within range of ±10%, formed the entire wafer. We selected amyloid beta (Aβ) peptides in the plasma of APP/PS1 transgenic mice as a study model and measured the peptide level by resistance changes of highly uniform rGO biosensor arrays. Aβ is a pathological hallmark of Alzheimer’s disease and its plasma concentration is in the pg mL^−1^ range. The sensor detected the Aβ peptides with ultra-high sensitivity; the LOD was at levels as low as 100 fg mL^−1^. Our results provide biological evidences that this wafer-scale high-resolution patterning method can be used in rGO-based electrical diagnostic devices for detection of low-level protein biomarkers in biofluids.

Reduced graphene oxide (rGO) has garnered significant attention as a promising nanomaterial because of its scalable production at low cost, high portion of chemically active sites, and solution processability^1^,^2^,^3^,^4^,^5^,^6^,^7^,^8^. High electrical conductivity and a large number of reaction sites are significant benefits of rGO-based biosensors for applications in clinical diagnostics of pathological biomarkers^9^,^10^,^11^. Because of these benefits, rGO-based biosensors are considered highly useful in rapid, disposable, and repeatable diagnostics at the point of care^12^,^13^. However, most of rGO-based biosensors have low fabrication yield and poor reliability due to the lack of uniform rGO patterning method in large areas. Thus, it is essential to develop rGO patterns enabling massive production with reliable reproducibility for simple and compact diagnostic instruments detecting biomarkers in low concentration.

Some mass production patterning methods have already been introduced: laser writing on assembled graphene oxide (GO) layers at a charged surface by repeating coating and washing steps^14^ or by spin coating^15^ and shadow-mask-assisted rGO spray-patterning for accurate patterns in large areas^16^. However, a large volume of GO solution was required and the thickness of the patterns was controllable only at the micron-scale. The surface-energy-engineered stamping method for patterning of the uniform rGO thin films prepared by a meniscus-dragging deposition (MDD) technique was introduced in our previous research^17^,^18^; the patternability of small patterns and arrays with dimensions on the order of several μm was demonstrated in a controllable ultra-thin rGO layer with remarkably high accuracy. Although our previous method forms the rGO micropatterns efficiently, the stamping method has some limitations, such as the directional dependency of striping caused by the molds; this is one of the critical obstacles to reproducible alignment during subsequent process steps such as photolithography. Cost-effective, high-yield fabrication methods are needed to ensure the practicality of graphene-based biosensing applications.

In this study, we introduce an effective wafer-scale rGO patterning method for biosensing applications based on dry etching in conventional micro-electro-mechanical systems (MEMS) equipment. Various shapes and sizes of rGO micro-patterns have been demonstrated through the optimization of patterning in conjunction with dry etching. The axis-homogeneity, which is calculated by analysing the electrical resistance of rGO patterns oriented in different directions on the wafer, was measured to verify the improved uniformity of patterning with dry etching. To assess biological applications of our rGO biosensors for precise detection of protein biomarkers in low concentration, wafer scale fabricated array of rGO biosensor were utilized as shown in Fig. 1. We collected blood of APP/PS1 transgenic Alzheimer mice and measured the plasma level of human amyloid beta (Aβ) peptides. The Aβ peptide is a blood-brain-barrier-penetrating biomarker of Alzheimer’s disease^19^,^20^ and requires highly sensitive detection methods because of its low molecular weight (4.5 kDa)^21^ and picomolar concentration in plasma. The Aβ peptides in plasma were detected to show the reliable immunoreaction response of the rGO biosensor through change of resistance with wafer scale fabrication.

## Results and Discussion

The major steps of the rGO patterning method are accomplished with conventional photolithography and dry etching processes to form accurate patterns with high yield at the wafer scale. First of all, the rGO thin film layer was deposited on the Si/SiO_2_ wafer. The rGO film was produced by the reduction of a GO layer that had been uniformly deposited by the MDD method^17^,^18^. The detailed process steps of the rGO deposition are presented in Fig. S1 of the Supporting Information section. The robust and uniform rGO film layer formed with MMD methods was treated the same as any other layer that is generally used in semiconductor fabrication and MEMS techniques. The sheet resistance of the deposited rGO layer across a 4-inch wafer was measured to demonstrate the high uniformity of the film formed by the MDD method; the results are summarized in Fig. S2(b). After the deposition of the rGO film, a photoresist (PR) layer was deposited on the rGO layer by spin coating as shown in Fig. S3. The photolithography, including exposure to ultra-violet (UV) light to form patterns on the PR layer, was performed with a commercial aligner. The patterned PR on the rGO layer was used as a sacrificial layer for selective etching of the rGO layer. The etching process was implemented with reactive ion etching (RIE), which is a kind of dry etching, in an oxygen atmosphere. In this way, the patterns were successfully transferred from the PR to the rGO layer on the SiO_2_ substrate.

**Figure 2:**
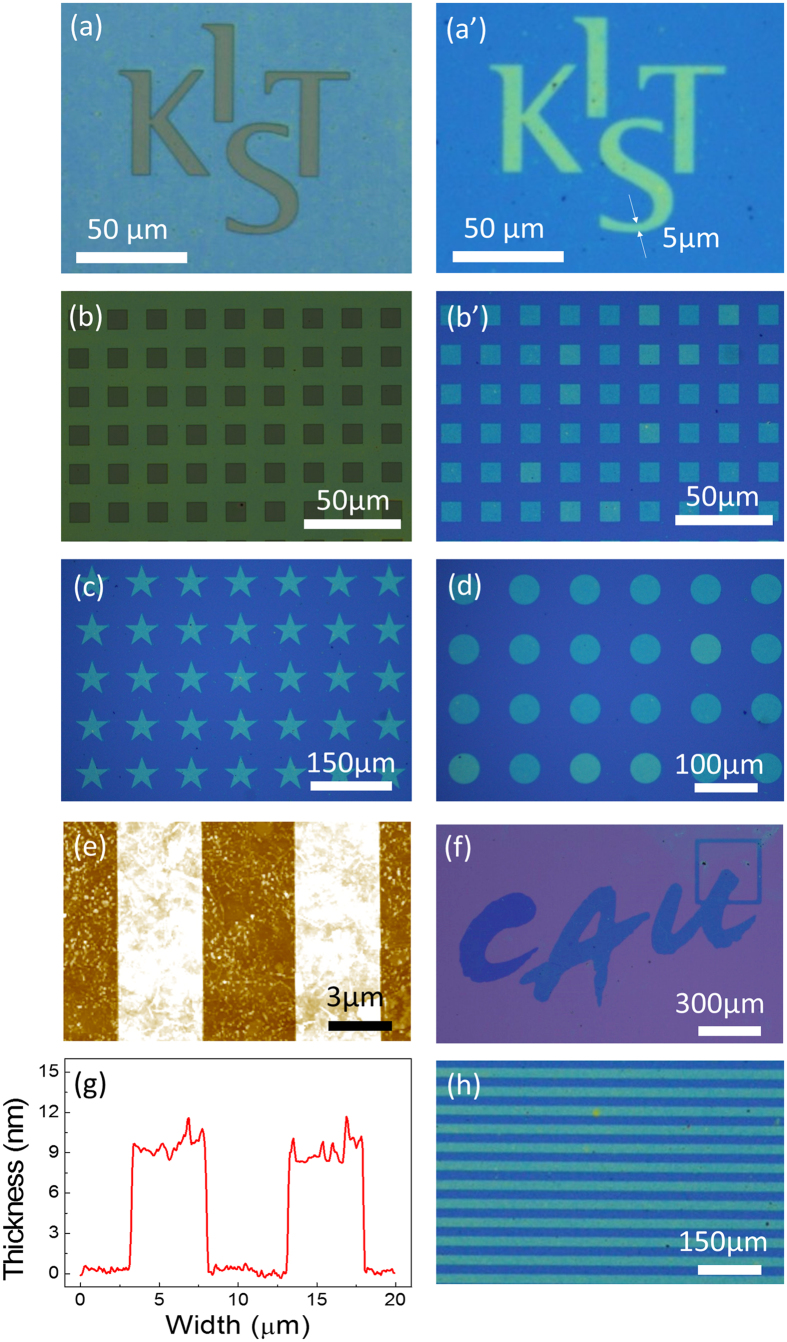
Various shapes of fabricated PR and rGO patterns. Optical images of (**a**) The ‘KIST’ logo PR patterns on the rGO layer (**b**) square array PR patterns. Optical images of rGO patterns on SiO_2_ substrate of (a′) ‘KIST’ (b′) square array (**c**) star (**d**) circle (**f**) logo of ‘CAU’ and (h) line. (**e**) AFM image of line patterns; and (**g**) analysis of the thickness of the line patterns. (The logos of ‘KIST’ and ‘CAU’ were permitted from each copyright holder to reproduce in this manuscript).

Various high-resolution micro-patterns were formed on the rGO to verify the transfer of the PR patterns to the rGO, as shown in Fig. 2. The optical images of Fig. 2a,b are the PR patterns of the logo ‘KIST’ and an array of rectangular patterns, respectively. Figure 2a′ show the optical images of the rGO patterns after the etching process. The rGO patterns are indistinguishable from the PR patterns. The PR functioned very effectively as a sacrificial-layer to transfer its own patterns to the rGO layer with high resolution and reproducibility.

The smallest width in the final rGO patterns in Fig. 2a′ was 5 μm as indicated by the white arrows. The square patterns of rGO in Fig. 2b′ were also obtained from the PR patterns through the etching process. The feature length of the single square pattern and the pitch size between the patterns are approximately 10 μm in the case of both the PR and the rGO as shown in Fig. 2b,b′, respectively. Other shapes such as stars, circles, the ‘CAU’ logo, and a pattern of parallel lines were also produced to show the patternability of various shapes with conventional dry etching. The array of 50-μm-wide single stars and the circular patterns with a radius of 25 μm were properly formed as shown in the optical images of Fig. 2c,d, respectively. The centre-to-centre distance between features was 100 μm in the arrays of squares and circles. In Fig. 2f, the ‘CAU’ logo was also reproduced within a 1.4 mm × 0.8 mm area, which is a relatively large area, with detailed expressions of the rough edges in each character. Patterns comprising 20-μm-wide lines with 20-μm spacing were also demonstrated as shown in Fig. 2h. As shown in the atomic force microscopy (AFM) image of Fig. 2e, narrow line patterns with 5-μm width and spacing were also fabricated and measured to verify the resolution of the patterning method. The thickness profile measured by AFM from the image of Fig. 2e is shown in Fig. 2g. The thickness of the rGO line patterns is about 8.7 nm, which is the expected value, based on the results of previously reported MDD studies^17^.

**Figure 3:**
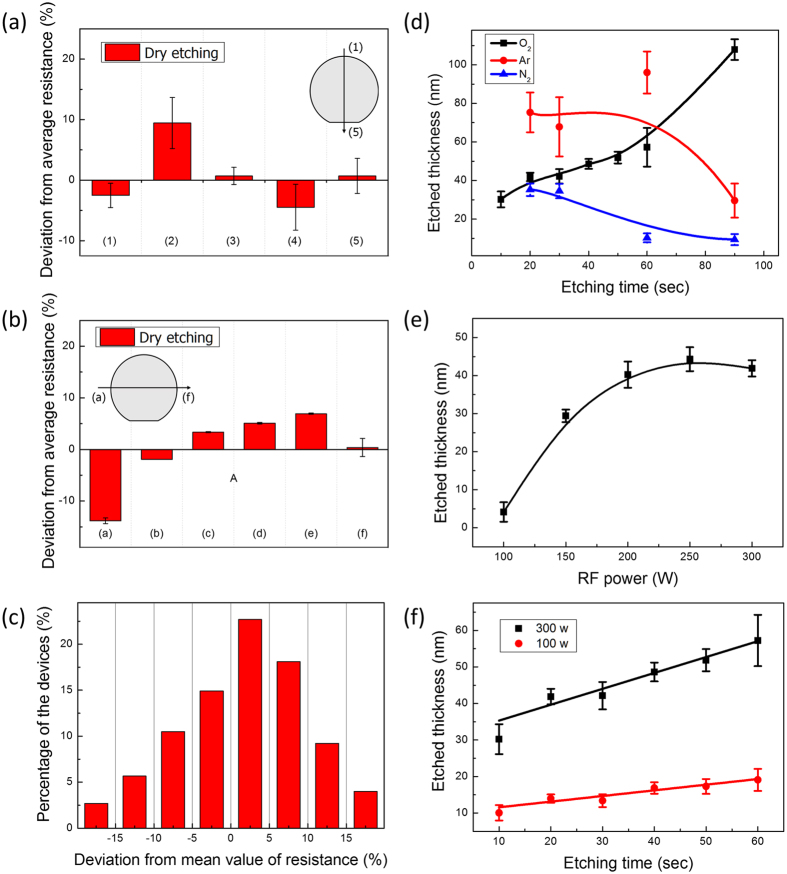
(**a–c**) Analysis of the deviations of the device resistance across the entire wafer, demonstrating the reliability and reproducibility of the dry etching patterning method. Deviation of resistance along (**a**) the y-axis, and (**b**) the x-axis. (**c**) Distribution of the deviations among a large number of devices. (**d**–**f**) Etched thickness of patterns as a function of (**d**) etching gas composition (O_2_: black line, Ar: red line, and N2: blue line) with 300 W RF power; (**e**) RF power in O_2_ gas for 30 s; and (**f**) time for 100 W (red line) and 300 W (black line) of RF power in O_2_ gas.

The resistance of fabricated rGO sensor devices was measured and analysed to show the uniform formation of rGO sensor arrays through patterning of rGO by dry etching across the entire 4-inch wafer. The detailed fabrication process is shown in Fig. S3. The variation of resistance in the rGO sensors as a function of position on the wafer was demonstrated as shown in Fig. 3a,b. The fabricated devices on the wafer and the detailed layout of the wafer are shown in Fig. S4. The average resistance (18.3 kΩ) of 24 rGO devices, each of which contains 24 individual arms (resulting in a total of 576 arms) was measured and set as a reference for the entire wafer. Each arm has 50-μm-wide and 100-μm-long rGO patterns as shown in Fig. S4(d). The deviation of resistance in each section from the average value was measured and calculated as a percentage.

The deviations along the y-axis, as shown by the red bars in Fig. 3a, were: −2.5 ± 2.0%, 9.4 ± 4.2%, 0.7 ± 1.4%, −4.5 ± 3.8%, and 0.7 ± 2.9% at sections (1), (2), (3), (4), and (5), respectively. The average values are derived from over 96 or 144 arms of rGO-based sensors in each section of the wafer. Resistance uniformity was better than 5%, except in section (2). Furthermore, the variation within each section is also quite low. The trend in deviations along the x-axis was similar to the y-axis, which shows that the dry etching method can achieve axis-homogeneous patterning. The deviation of resistance along the x-axis, as shown by the red bars in Fig. 3b, were: −13.8 ± 0.5%, −1.9 ± 0.1%, 3.3 ± 0.1%, 5.1 ± 0.1%, 6.9 ± 0.1%, and 0.4 ± 1.7% at sections (a), (b), (c), (d), (e), and (f), respectively. Most sections have around 5% of deviation from the average resistance, and the minimum variation in each section was 0.1%; this shows that the dry etching method can produce very uniform patterns throughout the entire wafer. The uniformity that was achieved here was higher than that reported by another group^22^, using a different method; and also higher than in our own previous study that utilized a polydimethylsiloxane (PDMS) stamping method^13^. A comparison of the uniformity and axis-homogeneity achieved by dry etching and by PDMS stamping is presented in Fig. S5.

The distribution of the deviations from the mean value of resistance was also analysed, as shown in Fig. 3c. The number of devices that had specific values of deviation was counted and calculated to display the percentages. The histogram shows that 66.2% of the rGO arms formed by dry etching were found within the deviation range of ±10% [range −20~−15%:2.7%, −15~−10%:5.7%, −10~−5%:10.5%, −5~0%:14.9%, 0~5%:22.7%, 5~10%:18.1%, 10~15%:9.2%, and 15~20%:4.0%, red bars of Fig. 3c].

To verify the uniformity of the pattern formation across the entire wafer, the thickness of the films produced by the dry etching method was measured as a function of the gas composition, the RF power of the plasma, and the etching time, as shown in Fig. 3d–f. The gases oxygen (O_2_), argon (Ar), and nitrogen (N_2_) were utilized at 300 W of RF power for dry etching by RIE to keep the etching times at reasonable levels. As shown in Fig. 3d, the O_2_ gas has a relatively linear etching profile that is sufficient to control etching thickness by controlling the etching time, whereas the other gases do not display this feature. To confirm the dependence of etching rate on RF power, the etched thickness in an O_2_ atmosphere after 30 s etching time was also measured, as shown in Fig. 3e. The etched thickness increased with increasing RF power in the range from 100 W to 200 W. Above 200 W of RF power, the etched thickness was not proportional to the power and then saturated, becoming almost independent of power. The etching time in an O_2_ atmosphere was also varied at two different fixed power levels to optimize the etching conditions. The etched thickness at 100 W and 300 W of RF power, which are, respectively, the lowest and highest values studied, was measured as a function of the etching time as shown in Fig. 3f. The etched thickness was linearly proportional to the etching time (from 10 to 60 s) with an etch rate of 0.43 nm/s at 100 W RF power. The etch rate of 300 W RF power was 2.3 nm/s; this is a higher value than the etch rate at 100 W. Thus, the controllable conditions of RF power and etching time in an O_2_ atmosphere could be adjusted to produce the desired thickness of target materials. The properties of the dry-etched rGO thin films were further characterized by their Raman and XPS spectra, as shown in Figs S6–S8.

Finally, the detection of Aβ peptide was demonstrated to show the reliable characteristics of the rGO-based sensors that had been fabricated on 4-inch wafers. The reactions of Aβ antibody and Aβ were detected through changes of resistance in the rGO sensor. The change of resistance is defined by Equation (1),


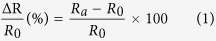


where ∆R, R_0_, and R_a_ are the absolute values of resistance changes, the initial resistance of the rGO, and the resistance after immobilization of antibodies, respectively.

**Figure 4:**
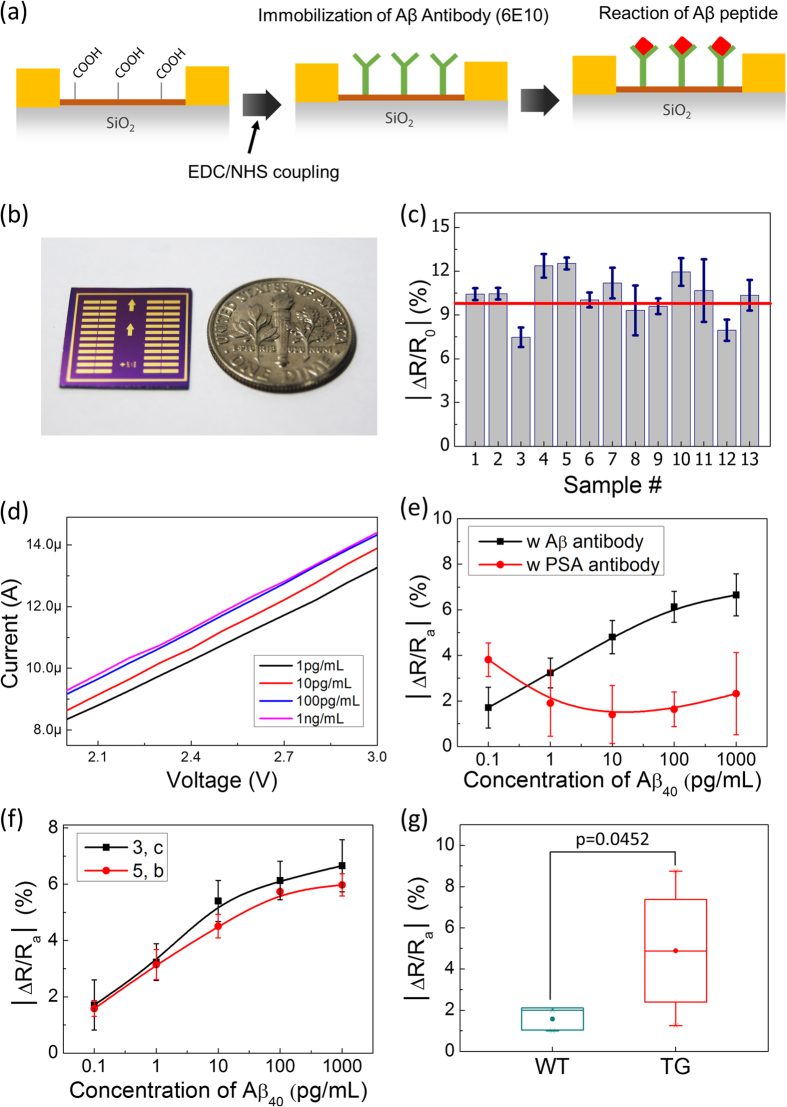
Demonstration of biosensing with an rGO-based sensor for Aβ_40_ detection (**a**) Sensing scheme based on Aβ_40_ and antibody (6E10) of Aβ_40_ (detection of the resistance changes due to the reaction of Aβ_40_ with immobilized antibody on the rGO surface); (**b**) a single fabricated device; (**c**) resistance changes from uniformly immobilized antibody at randomly selected devices (Number of devices: 13, number of arms in single device: 24; the red line shows the average of total changes of resistance); (**d**) current changes at applied voltage [2.0 V to 3.0 V] according to the concentration of Aβ_40_ (**e**) selective reaction of Aβ_40_ [resistance changes according to the concentration of Aβ_40_ were compared between the reaction with Aβ antibody (black line) and antibody for PSA (red line)]; (**f**) dynamic range of the biosensor, resistance changes according to the concentration of Aβ_40_ (from 100 fg mL^−1^ to 1 ng mL^−1^) with devices from (3, c) (black line) and (5, b) (red line) (**g**) discrimination test between WT (n = 3) mice and TG mice (n = 3). (Significant at p = 0.0452).

As shown in in Fig. 4a, the antibody for Aβ (6E10) was immobilized at carboxyl reaction sites of rGO by coupling with Ethyl-(dimethylaminopropyl) carbodiimide (EDC) and N-Hydroxysuccinimide (NHS). After the immobilization, the Aβ_40_ was reacted with the immobilized antibody on the rGO surface as an immunoassay reaction. The device used for this demonstration is shown in Fig. 4b. To verify the reproducibility of the stable immobilization of antibodies on the rGO surface, the measurement of resistance changes was repeated with 13 devices which were randomly selected from the same wafer as shown in Fig. 4c. The average value change in resistance [the red line in Fig. 4c] for 13 devices was 10.4 ± 1.4%. The values of each bar were acquired from a single device with 24 resistance-sensitive arms. Therefore, the data in Fig. 4c represent a total of 312 resistance measurements, which demonstrate the excellent uniformity of the response of the rGO sensors (from the same wafer) to immobilization of Aβ antibodies.

After the uniform immobilization of antibodies, the relative resistance changes (∆R/R_a_, %) resulting from the reaction of Aβ_40_ with its antibody was plotted as a function of the Aβ_40_ concentration to characterize the sensor as shown in Fig. 4d,e. The resistances were calculated with Ohm’s law from measurement of current according to the applied voltages (from 2.0 V to 3.0 V) as shown in Fig. 4d. The absolute values of ∆R/R_a_ in Fig. 4e–g were derived by replacing R_0_ with R_a_ and R_a_ with R_Aβ_ in equation (1), where R_Aβ_ is the absolute resistance after the reaction with Aβ_40_.

The resistance change was proportional to the Aβ_40_ concentration in the range of 100 fg mL^−1^ to 100 pg mL^−1^ as shown in the black line of Fig. 4e. The limit of detection (LOD) of Aβ is approximately 100 fg mL^−1^, which shows that the rGO sensor has ultra-high sensitivity. The test of selectivity was also tried with the antibody of Prostate-specific antigen (PSA) as shown by the red line of Fig. 4e. The PSA antibody was also immobilized at rGO surface with the EDC and NHS coupling. After the immobilization of the PSA antibody, Aβ_40_ was also applied to react with the PSA antibody. The resistance changes had no discernible dependence on the concentration of Aβ_40_ in the case of the PSA antibody. Each point of Fig. 4e was acquired from 12 arms of rGO, resulting in consumption of 120 arms for the experiments; 60 arms were used for each of the conditions. To show the more practical usage of wafer scale-fabrication for biosensing applications, the response of the Aβ_40_ reaction with randomly selected devices in the same wafer was also measured as shown in Fig. 4f. The devices were from positions (3, c) and (5, b) which are relatively close to the middle and edge parts of the wafer, respectively. The percentage of ∆R/R_a_ was measured with both devices, and the values tracked each other very closely as the concentration of Aβ_40_ increased in the range from 100 fg mL^−1^ to 100 pg mL^−1^. The ∆R/R_a_ was measured by sequentially applying Aβ_40_ from lower to higher concentration. Next, we measured levels of human Aβ peptides in mouse plasma samples to show the reliable working of the rGO biosensor with real samples as shown in Fig. 4g. We collected blood plasma of 7-month-old male transgenic (TG, n = 4) mice, with human Aβ peptides, as an Alzheimer patient model; their age-matched male wild type (WT, n = 3) mice were used as a control sample. The number of consumed devices per individual mouse was 1, resulting in utilizing 7 devices and 168 arms. The plasma of each mouse was reacted with immobilized antibody the same as in the previous recombinant Aβ reaction. Each group of mice has a different value of resistance changes and the test discriminated with high significant value (p = 0.0452) as shown in Fig. 4g.

## Conclusion

In summary, the effective wafer-scale rGO patterning was accomplished by adapting the highly reliable and reproducible dry etching techniques that are generally used in MEMS or semiconductor fabrication method for biosensors. The gold electrode was also aligned and formed on the rGO patterning with conventional photolithography and lift-off, thanks to the well-formed rGO layer created by the MDD method. Most existing rGO-based devices have an rGO layer only at the outermost layer after forming the other structures, such as electrodes. These results also show that our method can be applicable in various fields that need subsequent fabrication steps after forming the rGO patterns. The resistance changes by the reaction of Aβ_40_ and Aβ antibody in rGO sensors, which were fabricated at wafer-scale, were also reproducible because of the reliable rGO patterning method with ultra-high sensitivity (LOD ≈ 100 fg mL^−1^). This level of performance is sufficient to permit the utilization of the sensor in the diagnosis of Alzheimer’s disease^19^. We hope that widespread usage of graphene-based biomolecule detectors will become feasible with our fabrication method. For that, we would also try to acquire more intense reactions by treatment of the rGO and then be able to detect biomolecules from actual clinical samples in biofluids such as human blood and urine which is the one of the most convenient media for diagnosis.

## Methods

### Deposition of rGO thin films on 4-inch wafers

Detailed information is described in the Supporting Information section in Fig. S1.

### Patterning of rGO

The PR (GXR601, AZ electronic materials) was deposited by spin coating at 3000 rpm for 30 s on the rGO film layer that had been formed by the MDD method to obtain approximately 1 μm of thickness. Photolithography was performed, using a commercial aligner (MA6 Mask aligner, Karl Suss) with 15 mW mm^−2^ of UV light exposure for forming the sacrificial pattern of PR. The coated substrate was pre-baked at 100 °C for 90 s before developing with developer (AZ^®^ 300 MIF, AZ electronic materials). Hard-baking at 115 °C for 60 s was also utilized to ensure a robust sacrificial layer during the etching process. RIE (Advanced Vacuum & STS, VISION 320 RIE) was applied in an O_2_ atmosphere with the RF power and time optimized according to the expected etched thickness to produce the rGO patterns on the SiO_2_ substrate. After the etching, the remaining PR was removed with acetone, and the substrate was sequentially washed with methanol, isopropyl alcohol, and deionized (DI) water. The etched thickness was measured with an AFM (Park systems, XE-100, Korea).

### Fabrication of rGO biosensor

Detailed information is introduced in the Supporting Information section in Fig. S3.

### Electrical measurements

The sheet resistance of the rGO films was measured by the four-point-probe method with a Keithley 2400. The resistance changes were recorded with a semiconductor analyzer (Agilent, B1500A) and probe station (Unitek corp., AP-8000, Korea).

### Antibody immobilizations and Aβ_40_ reaction

Both 6E10 monoclonal antibody (Covance, USA, 1 mg mL^−1^) for Aβ and PSA antibody (Fitzgerald, USA, 1 mg mL^−1^) were diluted to a concentration of 10 μg mL^−1^ with 0.1X phosphate-buffered saline (PBS) solution. After the dilution, each antibody was immobilized at the rGO surface with 2 mM of EDC and 8 mM of NHS for 2 h. Washing for removing residual materials was accomplished with 0.1X PBS and DI water. The recombinant Aβ_40_, which has 1–40 amino acids was diluted in 10 mM PBS buffer to get a target concentration from 100 fg mL^−1^ to 1 ng mL^−1^. The diluted recombinant Aβ_40_ was dropped on the antibody-immobilized rGO surface and reacted for 20 min. After the reaction, the surface was washed with PBS buffer and DI water.

### Preparation of transgenic mice

Double APP/PS1 transgenic mice (TG, strain name: B6C3-Tg (APPswe, PSEN1De9) 85Dbo/J) and their WT mice (B6C3F1) were purchased from Jackson Laboratory (Bar Harbor, Maine, USA). Expression of both genes, APP and PSEN1, was confirmed before the experiment began via a PCR instrument from Bio-Rad (S1000 Thermal-Cycler) using the standard PCR condition from Jackson Laboratory, the PCR-remix provided by Cosmo-Genetech (G-taq PCR premix kit, CMT-6002), and DNA from mice tails. The subject male mice (TG and WT; n = 4 and n = 3, respectively) were 7 months of age at the time of blood collection, and they were housed in a facility under controlled temperature, with an alternating 12 h light-dark cycle and access to food and water *ad libitum*. All animal experiments were carried out in accordance with the National Institutes of Health guide for the care and use of laboratory animals (NIH Publications No. 8023, revised 1978). The animal studies were approved by the Institutional Animal Care and Use Committee of Korea Institute of Science and Technology.

## Additional Information

**How to cite this article**: Kim, J. *et al*. Wafer-scale high-resolution patterning of reduced graphene oxide films for detection of low concentration biomarkers in plasma. *Sci. Rep.* **6**, 31276; doi: 10.1038/srep31276 (2016).

## Supplementary Material

Supplementary Information

## Figures and Tables

**Figure 1:**
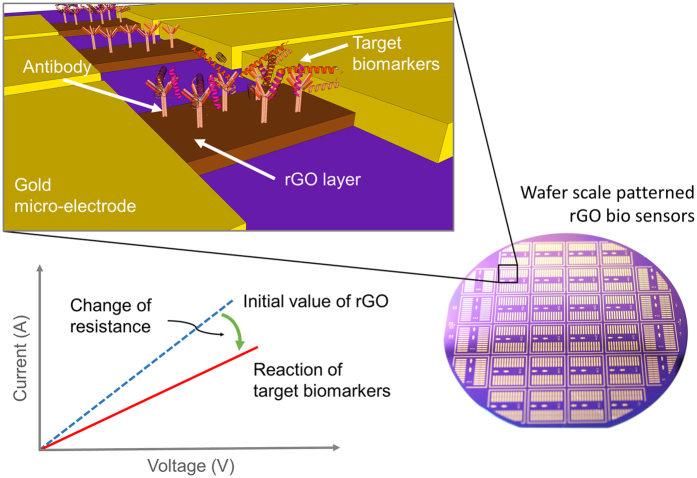
Schematic view of wafer-scale fabricated rGO biosensors (array type of rGO biosensor). Immunoreaction of target biomarkers with the antibody was detected by measuring changes of resistance at the rGO layer. The antibody was immobilized at the rGO layer which acts as a sensing zone. The rGO layers were located between the gold micro-electrodes.
