# Supplementary material for: Wafer-scale high-resolution patterning of reduced graphene oxide films for detection of low concentration biomarkers in plasma
Source: Sci Rep. 2016 Aug 10;6:31276. doi: 10.1038/srep31276 (PMC4978995; doi:10.1038/srep31276)
Supplement: Supplementary Information [file srep31276-s1.pdf]

Supporting Information

Wafer-scale high-resolution patterning of reduced graphene oxide films for detection of low concentration biomarkers in plasma

Jinsik Kim[a], Myung-Sic Chae[a], Sung Min Lee[b], Dahye Jeong[a], Byung Chul Lee[a], Jeong Hoon Lee[c], YoungSoo Kim[d], Suk Tai Chang[b] and Kyo Seon Hwang[a]

[a] Center for BioMicrosystems, Korea Institute of Science and Technology, Seoul 136-791, Korea

[b] School of Chemical Engineering and Materials Science, Chung-Ang University, Seoul, Korea

[c] Department of Electrical Engineering, Kwangwoon University, 447-1 Wolgye, Nowon, Seoul 01897, Korea

[d] Convergence Center for Dementia, Korea Institute of Science and Technology, Seoul 136-791, Korea

Preparation of rGO thin films

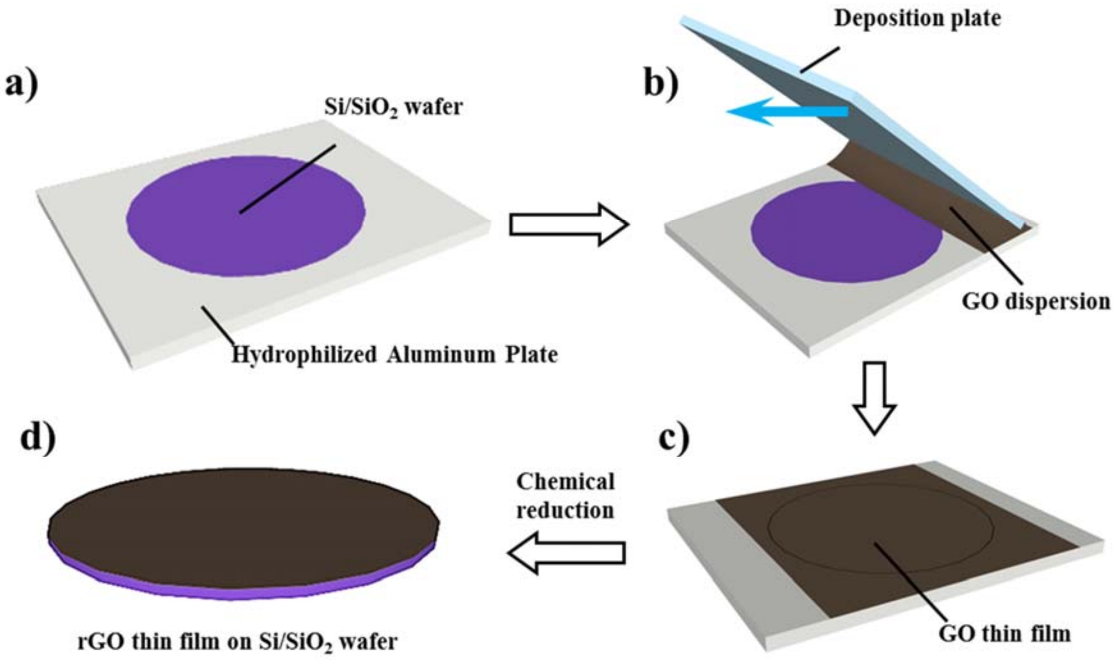

Figure S1. Schematic of the rGO coating process by the MDD method. (a) Place a 4-inch Si/SiO_2 wafer into a recess formed in an aluminum plate; the depth of the recess equals the thickness of the wafer. (b) Inject GO solution into the wedge between the deposition plate and the substrate, followed by dragging the deposition plate to form a uniform GO thin film on the wafer. (c) Dry the as-coated GO thin film on a hot plate at 100 °C. (d) Chemical reduction of the GO film by HI acid vapor produces a highly uniform rGO thin film.

The coating process of the highly uniform rGO thin films is illustrated in Figure S1. The uniform rGO thin films on Si/SiO_2 wafers were fabricated by applying the meniscus dragging deposition (MDD) technique.[1] An aqueous GO solution was acquired from Graphene Supermarket (SKU-HCGO-W-175). The GO dispersion was mixed with ultrapure Milli-Q water to obtain a GO coating solution with a concentration of 3.25 mg/mL. A glass plate and 4-inch Si/SiO_2 wafer were

used as a deposition plate and coating substrate. The glass plate and the wafer were hydrophilized with a piranha solution for 30 min and thoroughly rinsed with DI water. A recess with the same size and shape as the wafer was formed in an aluminum plate; the depth of the recess was equal to the thickness of the wafer. The aluminum plate was hydrophilized by exposure to UV-Ozone (Ozone cure 16, Minuta Technology Co.). After inserting the wafer into the hole in the aluminum plate, the glass deposition plate was placed in contact with the coating the wafer at an angle of 30°. An 800 μL drop of the GO coating solution was injected into the wedge between the glass deposition plate and the Si/SiO_2 wafer. The deposition plate, connected to a motorized stage (AL1-1515-3S, Micro Motion Technology), was pushed with an alternating motion at a constant speed of 20 mm/s. The resulting GO films were dried on a hot plate at 100 °C. The dried GO films were reduced using hydriodic (HI) acid vapor at 80 °C for 1 h to obtain highly uniform rGO thin films.

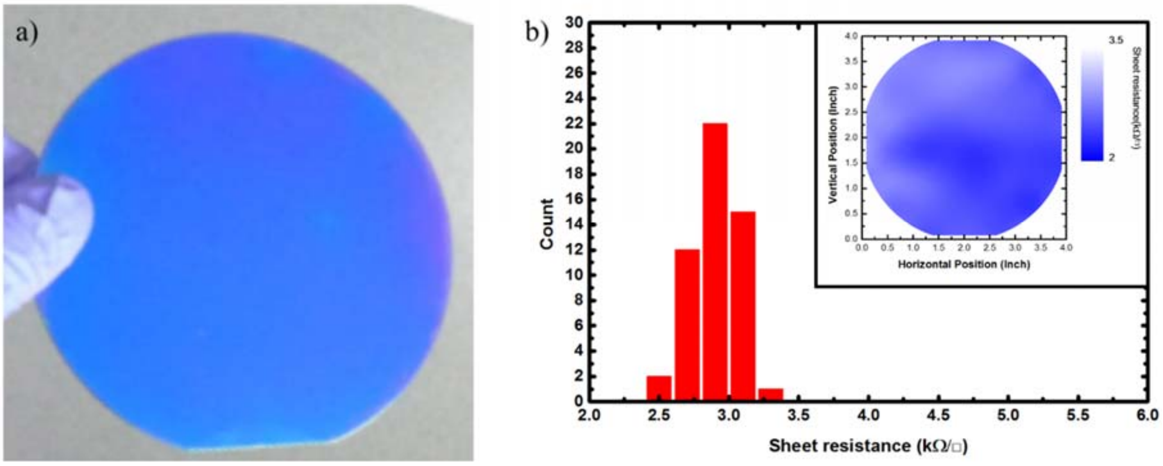

Figure S2. (a) Optical image of the rGO thin film on the 4-inch Si/SiO_2 wafer. (b) Statistical summary of sheet resistance of the 4-inch rGO thin film prepared by the MDD method. The inset in (b) corresponds to the actual spatial distribution of the sheet resistance in the rGO thin film.

Fabrication of rGO based biosensor

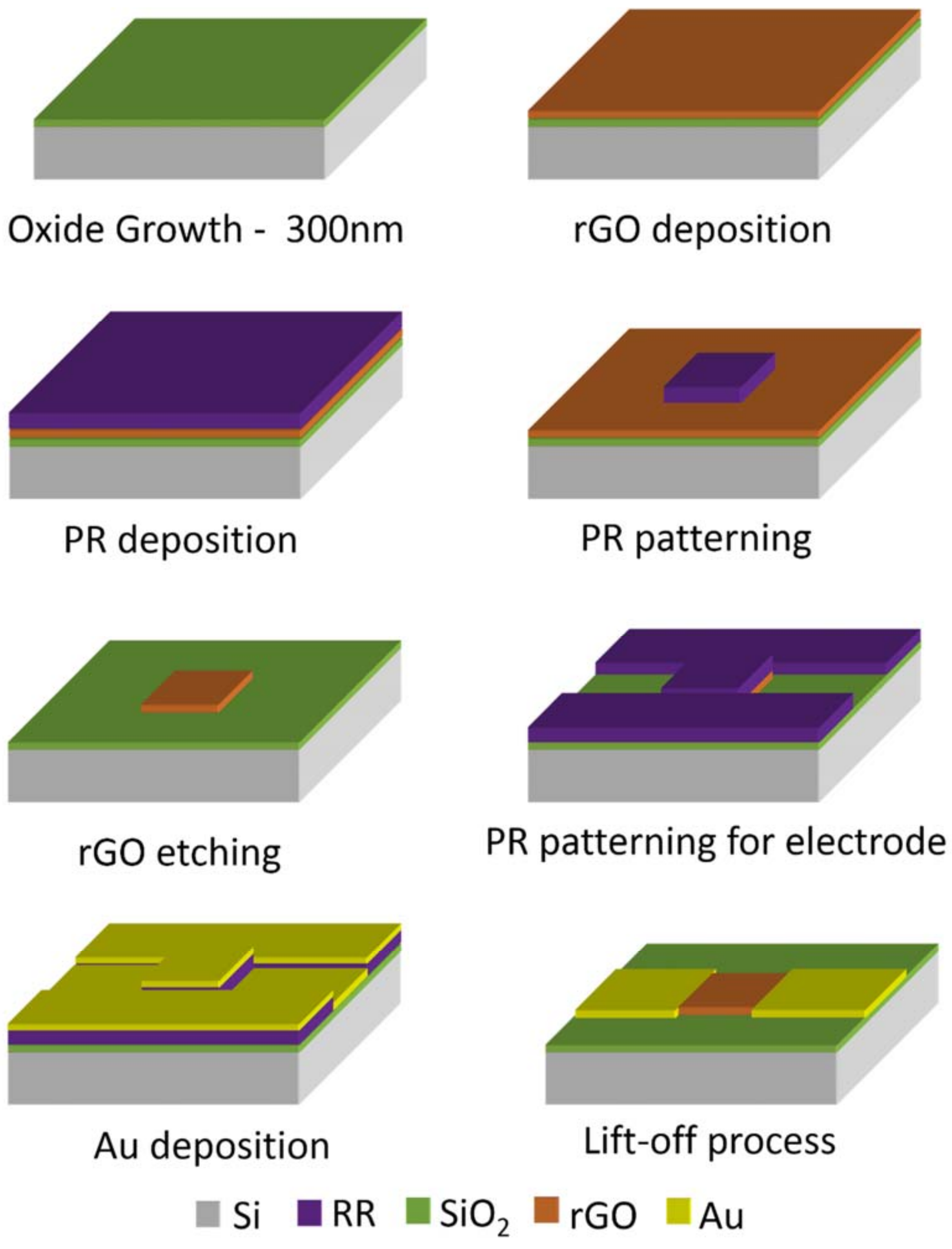

Figure S3. Fabrication process of the rGO based biosensor.

A Si substrate with 300 nm of thermally grown SiO_2 was used. The patterning of rGO was the same as described in the experimental section of the manuscript. The rGO layer was deposited by

the MDD method and patterned with conventional photolithography and RIE etching after the PR deposition and patterning. After the rGO patterning with RIE etching, PR (DNR-L300-30, Dongjin Semichem Co., Korea) was deposited to form electrodes. The spin coating method was employed at 3000 rpm for 30 s to form the PR layer. The target thickness of PR for the electrode was 2 μm. Soft baking at 90℃ for 100 s was applied before the alignment and exposure to UV light (15 mW/mm^2). A commercial aligner (MA6 Mask aligner, Karl Suss) was also used to align with the rGO patterns. Diluted developer (AZ® 300 MIF, AZ electronic materials) with DI water at a 1:1 ratio was used to form the electrode patterns. As shown in Figure S2 of "Au deposition", the gold was deposited on the PR patterns by an e-beam evaporator (ULVAC, ei-5) at a 0.1 nm/s deposition rate for a 200-nm-thick Au layer. The completely gold-coated substrate was immersed in acetone for 1 h to remove the PR by a lift-off process. In this manner, rGO patterns with gold contacts for biological applications were fabricated. The biomolecules can be immobilized in the rGO patterns as a sensing zone and detected by means of the resistance changes.

Wafer level fabrication of rGO based biosensor

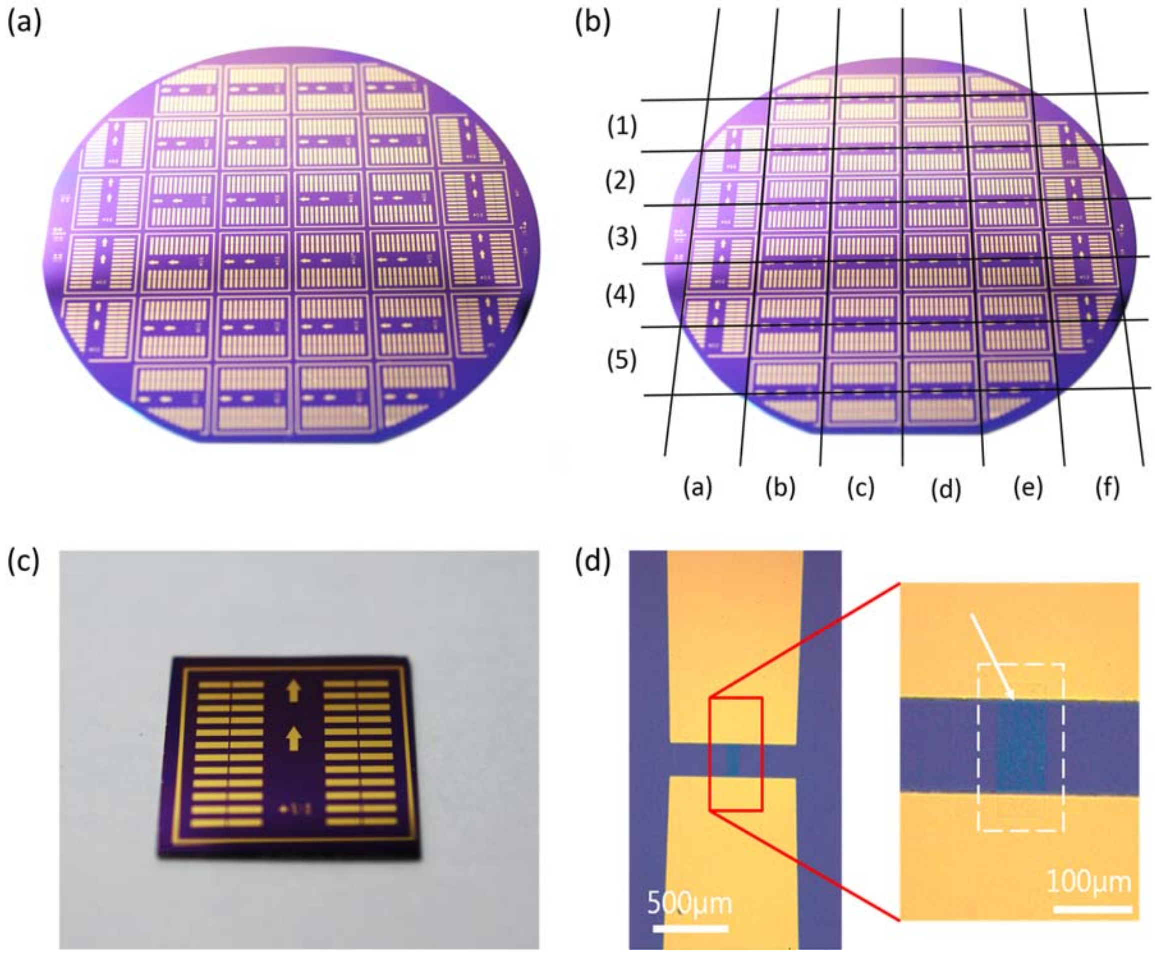

Figure S4. Images of the fabricated wafer. (a) Entire wafer; (b) sectioning of wafer for analysis; (c) a single device; (d) a single electrode-arm pair (the magnified image from red rectangular area shows the rGO pattern which is connected with gold electrodes. The rGO pattern located in white dashed rectangular)

The fabricated wafer is shown in Figure S4 (a). The 5 sections from (1) to (5) along the y-axis and 6 sections from (a) to (f) along the x-axis of the wafer were defined as shown in figure S4 (b). Each section has the same dimensions, and contains a single device that has 24 electrode-arm pairs with rGO sensing zones as shown in Figure S4 (c). The fabricated rGO layer (width: 50 μm, height:

100 μm) which acts as the sensing zone between a single electrode-arm pair is also shown in Figure S4 (d). The white arrow in Figure S4 (d) indicate the overlapped layer of rGO and gold electrode. The rGO patterns were well-formed and connected with electrodes with excellent alignment.

Uniformity-analysis of rGO patterns in 4 inch wafer

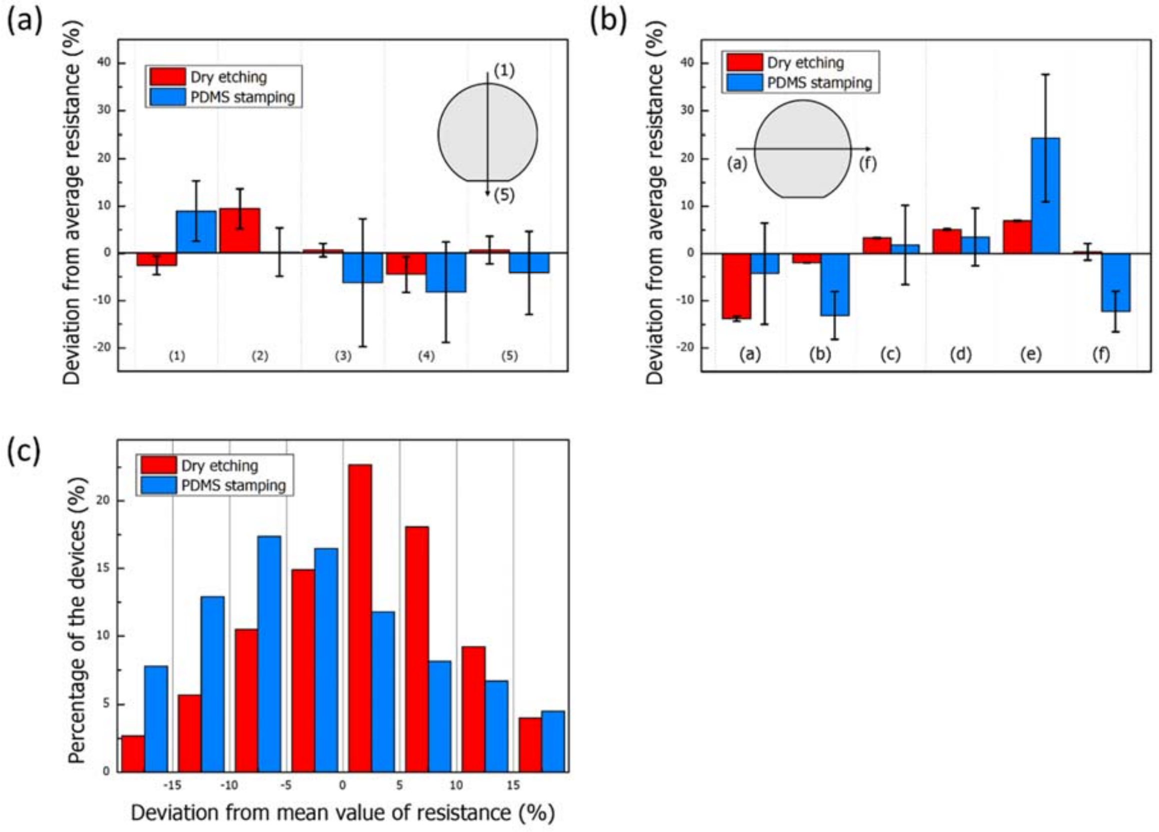

Figure S5. Comparison of the deviations in resistance throughout the entire wafer for devices fabricated by dry etching and by PDMS stamping, to demonstrate the reliability of the patterning method and the axis homogeneity. Deviation of resistance is presented along (a) the y-axis and (b) x-axis. (c) Distribution of resistance deviations of the devices (red bars: dry etching, blue bars : PDMS stamping).

The dry etching patterning method and the PDMS stamping method that had been introduced in our previous paper were compared to show the axis-homogeneous wafer scale patternability of dry etching. The devices fabricated with PDMS stamping were produced with the same processes

shown in Figure S3, but without the rGO patterning methods. The average value of resistance in all the devices on the wafer was 18.3 ± 0.4 kΩ in both cases.

The average resistance values along the y-axis of the rGO sensor arm that was fabricated with the PDMS stamping method were 8.9 ± 6.3%, 0.3 ± 5.1%, -6.2 ± 13.5%, -8.2 ± 10.6%, and -4.1 ± 8.8% at (1), (2), (3), (4), and (5), respectively [blue bars in Figure S5 (a)]. The deviation of resistance in the sections along the x-axis denoted as (a), (b), (c), (d), (e), and (f) were 4.2 ± 10.7%, -13.1 ± 5.0%, 1.8 ± 8.4%, 3.5 ± 6.0%, 24.3 ± 13.4%, and -12.3 ± 4.3% in the case of patterning with PDMS stamping as shown by the blue bars in Figure S5 (b). Although the deviations were reasonably low in both cases, the dry etching method resulted in significantly more uniform resistance throughout the entire wafer. The center sections such as (b), (c), and (d) have more uniform average resistance than the side sections. The smaller variations from average resistance by the dry etching method were also demonstrated in the analysis along the x-axis. Although the fluctuation of resistance values along the x-axis were higher than along the y- axis in the case of PDMS stamping, the deviation values of the x-axis and the y-axis were approximately the same in the patterns produced by dry etching; this shows that the dry etching method can accomplish axis-homogeneous patterning.

The distribution of deviations from the average resistance (18.3kΩ) for dry etching compared to PDMS stamping was also analyzed as shown in Figure S5 (c). The result of the PDMS stamping method was that 53.8% of the rGO arms had deviations within ±5% of the average [range -20 ~ -15%: 7.8%, -15 ~ -10%: 12.9%, -10 ~ -5%:17.4%, -5 ~ 0 %: 16.5 %, 0 ~ 5 %: 11.8%, 5 ~ 10%: 8.2%, 10 ~ 15%: 6.7% and 15 ~ 20%: 4.5%, blue bars of Figure S5 (c)], resulting in a lower fabricating yield than the dry etching method.

Analysis of etched rGO patterns: Characterization of the rGO thin films by Raman and XPS spectra

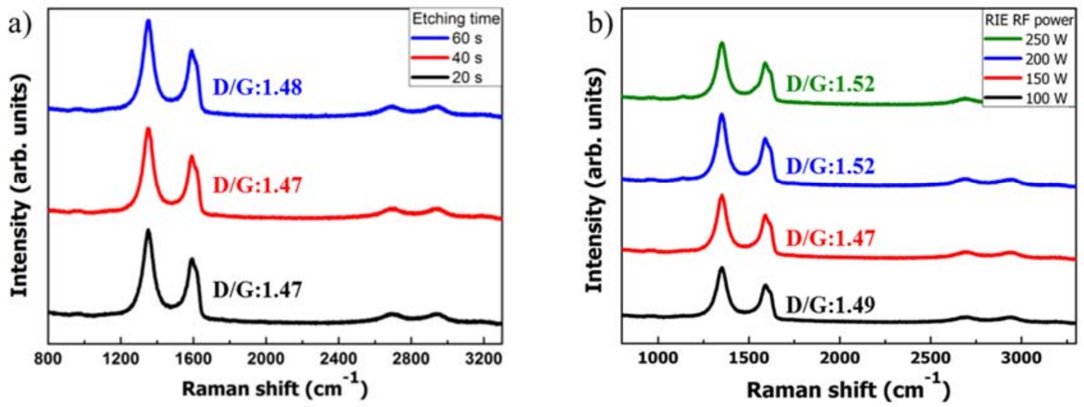

Figure S6. Raman spectra of the patterned rGO films by dry etching (a) for various etching times (black line: 20 s, red line: 40 s, blue line: 60 s) and (b) for various RIE RF powers (black line: 100 W, red line: 150 W, blue line: 200 W, green line: 250 W)

To examine the structural destructive effect of the patterning process, Raman spectroscopy and X-ray Photoelectron spectroscopy (XPS) were carried out. The Raman spectra were obtained by an inVia™ Raman microscope (Renishaw) with a 532 nm laser source, and the XPS spectra were obtained by K-alpha (Thermo VG, UK) under a vacuum of 4.8 x 10^{-9} mbar. For graphene-based materials, such as rGO, there exist characteristic peaks around 1350 cm^{-1}, 1600 cm^{-1}, and 2700 cm^{-1} that are called the D peak, G peak, and 2D peak, respectively.[2-4] The ratio of the D peak to the G peak (the D/G ratio) is one of the parameters that is widely used for analyzing structural defects of graphene. Similar Raman spectra were obtained in the dry-etched rGO films with different etching times with no significant increase in the D/G ratio and no shift of the characteristic peak positions, as shown in Figure S6 (a); this indicates that the etching process did not cause any significant damage to the rGO films.

Similarly, no significant increment of the D/G ratio in the Raman spectra of the rGO films treated with the increased RF power during RIE was observed as displayed in Figure S6 (b). Therefore, the high-resolution micro-patterning of the rGO thin films with the dry etching method can be achieved without significant damage to the resulting rGO patterns for biosensor applications.

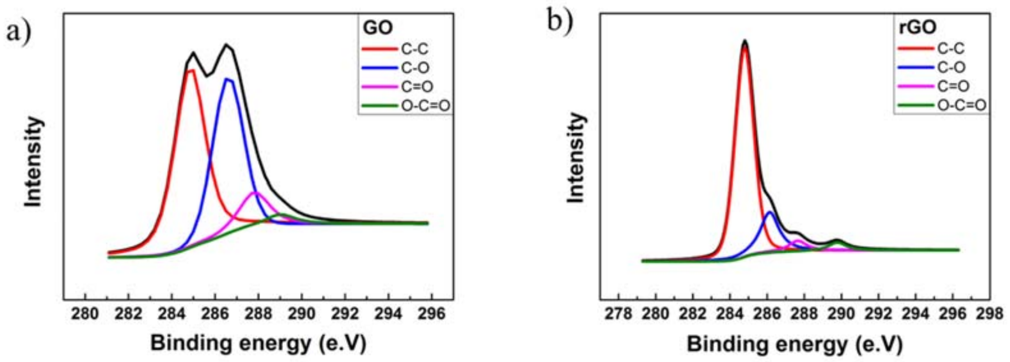

Figure S7. XPS spectra of films of (a) GO and (b) rGO prepared by the MDD method.

Table 1. Bonding ratio

	C-C	C-O	C=O	O-C=O
GO	**0.49**	**0.37**	**0.10**	**0.04**
rGO	**0.74**	**0.18**	**0.04**	**0.04**

For additional characterization of the rGO film, XPS analysis was also carried out. XPS can detect binding energy of electrons. The deconvoluted C 1s XPS spectra obtained from the GO and rGO thin films formed by the MDD method are shown in Figure S7. The C 1s peak of the films was separated into one main C-C and three small C-O components: C-C bonds (~284.8 eV), C-O (~286.2 eV), C=O (~287.8 eV), and O-C=O (~289 eV).[5,6] The most notable feature after reduction of the GO film is the increase of C-C bonding ratio in the C 1s spectra from 49% (GO) to 74%

(rGO) as shown in table 1. In addition, the peak intensities and the atomic ratios (O 1s/C 1s) of the rGO film were reduced in comparison with those of the GO film, which indicates that the reduction of the GO film was performed effectively by the HI acid vapor treatment.

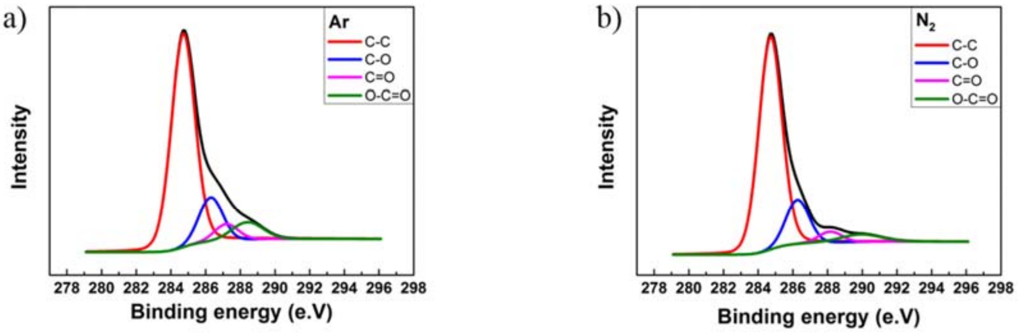

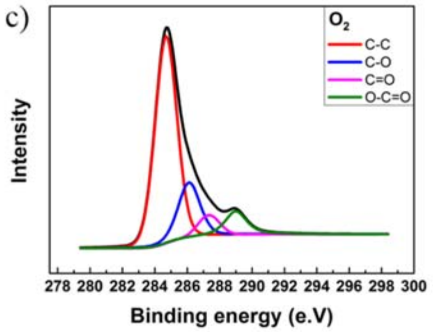

Table 2. Binding ratio

	C-C	C-O	C=O	COO
Ar	0.73	0.14	0.05	0.07
N2	0.77	0.15	0.04	0.04
O2	0.65	0.19	0.06	0.09

Figure S8. XPS spectra of rGO films after dry etching in various plasma gas compositions with (a) Ar, (b) N_2, and (c) O_2 gases. (RF power: 100 W, etching time: 20 s)

XPS spectra of the rGO films that had been dry-etched in different gas environments (Ar, N_2, O_2) are shown in Figure S8. The ratio of C-C bonds in the C 1s spectra of the rGO film treated in the O_2 atmosphere is decreased to 65%, while the rGO films in the Ar and N_2 gases show similar values to that of the as-prepared rGO film, because of the activated oxygen induced during the RIE etching in the O_2 atmosphere.

[1] Y. U. Ko, S.-R. Cho, K. S. Choi, Y. Park, S. T. Kim, N. H. Kim, S. Y. Kim, S. T. Chang, *Journal of Materials Chemistry* 2012, *22*, 3606.
[2] M. S. Dresselhaus, A. Jorio, M. Hofmann, G. Dresselhaus, R. Saito, *Nano Lett.* 2010, *10*, 751.
[3] F. Tuinstra and J.L. Koenig, *The Journal of Chemical Physics* 1970, *53*, 1126.
[4] Y. y. Wang, Z. h. Ni, T. Yu, Z. X. Shen, H. m. Wang, Y. h. Wu, W. Chen, A. T. Shen Wee, *The Journal of Physical Chemistry C* 2008, *112*, 10637.
[5] H. A. Becerril, J. Mao, Z. Liu, R. M. Stoltenberg, Z. Bao, Y. Chen, *ACS Nano* 2008, *2*, 463.
[6] S. V. Tkachev, E. Y. Buslaeva, A. V. Naumkin, S. L. Kotova, I. V. Laure, S. P. Gubin, *Inorganic Materials* 2012, *48*, 796.
